# Preventative Management of Sepsis-Induced Acute Respiratory Distress Syndrome in the Geriatric Population

**DOI:** 10.7759/cureus.34680

**Published:** 2023-02-06

**Authors:** Elizabeth Geyer-Roberts, Diana A Lacatusu, Jessica Kester, Gina Foster-Moumoutjis, Mojda Sidiqi

**Affiliations:** 1 Department of Medicine, Nova Southeastern University (NSU) Dr. Kiran C. Patel College of Osteopathic Medicine, Davie, USA; 2 Department of Family Medicine, Nova Southeastern University (NSU) Dr. Kiran C. Patel College of Osteopathic Medicine, Davie, USA

**Keywords:** geriatric, prevention, osteopathic, acute respiratory distress syndrome, sepsis

## Abstract

Sepsis and its treatment are the most common etiologies of acute respiratory distress syndrome (ARDS), which has a disturbing mortality rate. Sepsis management relies heavily on the introduction of resuscitative fluids. However, when fluids are paired with the circulating inflammatory mediators of sepsis, patients are prone to lung damage. Survivors of sepsis-induced ARDS become plagued with functional and/or psychological sequelae such as impaired memory, difficulty in concentrating, and decreased mental processing speed. Specific techniques can be implemented when diagnosing and treating elderly patients with sepsis to prevent the onset of ARDS, including bed elevation and early antibiotics. Additionally, albumin infusion may be beneficial; however, more research must be conducted. Finally, inflammatory mediators, including serum mannose biomarkers and extracellular histone therapy, show a promising avenue for future treatment. Although there is limited research on osteopathic manipulative medicine (OMT) on ARDS or sepsis-induced ARDS, OMT that focuses on alleviating rib and thoracic somatic dysfunctions has been used as an adjunct therapy to treat other respiratory diseases, such as pneumonia and chronic obstructive pulmonary disease (COPD). The results of these studies may garner interest in whether the use of OMT as an adjunct therapy may be beneficial for patients with ARDS or sepsis-induced ARDS. This paper is intended to review the current guidelines for sepsis and ARDS management in elderly patients to identify measures to prevent sepsis-induced ARDS.

## Introduction and background

Sepsis is a major public health concern leading to approximately 20% of all-cause global deaths [1]. It drastically impacts vulnerable populations such as neonates, the elderly, pregnant women, those with underlying health conditions, and the immunocompromised. Mortality depends on multiple factors such as timely diagnosis and quality of care, which includes health infrastructure, infection prevention, and clinical management [1]. Those who survive sepsis are faced with long-term health consequences, including physical and cognitive impairment, mental health disorders, and an overall increased mortality [1].

Over the last 10 years, sepsis has increased in global attention as world health leaders have worked to decrease alarming mortality rates. In 2016, the 2014/2015 global task force published new sepsis recommendations in the *Journal of the American Medical Association* (JAMA) [2]. Various medical summits have focused on sepsis management, including the 2014/2015 global task force, which published new sepsis recommendations in JAMA in 2016. In 2017, the 70th World Health Assembly proposed a resolution to improve sepsis prevention, diagnosis, and management. From that assembly, the World Health Organization released a 56-page Global report on the epidemiology and burden of sepsis. In 2021, international guidelines on sepsis and septic shock were published [3].

Before the 2014/2015 global task force, the last recommendations for sepsis were published in 2001. A global task force was created by the Society of Critical Care Medicine (SCCM) along with the European Society of Intensive Care Medicine (ESICM) in 2014, to address the need for updated guidance on sepsis. It consisted of four face-to-face meetings, where the goal was to discuss new recommendations for combating sepsis. In addition to new recommendations for sepsis, the task force also established a new definition of sepsis. Their report was published in JAMA in 2016 and has been endorsed by more than 30 medical societies from six continents [2]. According to the 2016 JAMA recommendations, sepsis is defined as a life-threatening organ dysfunction caused by a dysregulated host response to infection, where organ dysfunction is characterized by a Sequential Organ Failure Assessment (SOFA) score of 2 or more, which is associated with an in-hospital mortality greater than 10% [2]. Additionally, septic shock is defined as a subset of sepsis where profound circulatory, cellular, and metabolic abnormalities are associated with a greater risk of mortality than with sepsis alone [2].

Sepsis management relies heavily on the introduction of fluids. However, when fluids are paired with the circulating inflammatory mediators of sepsis, patients are prone to lung damage. Sepsis and sepsis treatment are the most common etiologies of acute respiratory distress syndrome (ARDS) [4]. ARDS is a blanket term for respiratory failure that often results in mechanical ventilation. ARDS is defined by the acute onset of noncardiogenic pulmonary edema, hypoxemia, and the need for mechanical ventilation [4]. Like sepsis, ARDS has a disturbing mortality rate of 30% to 40%, and those with sepsis-induced ARDS have a mortality rate greater than those with other risk factors [5]. Survivors of sepsis-induced ARDS become plagued with functional and/or psychological sequelae such as impaired memory, difficulty concentrating, and decreased mental processing speed [6].

Comorbidities predispose the elderly population (aged ≥65 years) to both sepsis and ARDS, leading to higher mortality and morbidity. The mortality rates of elderly patients with severe sepsis and septic shock are between 50% and 60%; this is 1.3 to 1.5 times higher than younger cohorts [7]. Similarly, the mortality rate for elderly patients with ARDS is also higher than in younger cohorts, where a study that compared 90-day mortality rates of patients found 30% mortality in young patients compared to 43% in elderly patients [8].

Increased mortality and morbidity in the elderly population necessitate the consideration of prophylaxis measures for sepsis-induced ARDS. With a particular focus on the elderly population and with consideration of both allopathic and osteopathic approaches, this paper reviews the current treatments of sepsis and identifies possible measures to prevent sepsis-induced ARDS.

The following search string was used in the PubMed database and filtered for studies published in the last 15 years: “((Sepsis) OR (Septic shock) OR (Acute respiratory distress syndrome)) AND ((Fluid management) OR (resuscitative fluids) OR (Corticosteroids) OR (Albumin) OR (Prevention) OR (Osteopathic)).” The studies included randomized controlled trials, meta-analyses, systematic reviews, and reviews published in the English language. Case studies and animal studies were excluded. Our criteria also excluded studies that did not focus on the relationship between sepsis and ARDS and those that did not include the elderly in the study cohort. The initial search yielded 8,387 results, and of these, 41 articles were selected and included in this study.

## Review

Guidelines for sepsis management 

According to the 2021 International Guidelines for the Management of Sepsis and Septic Shock [3], at least 30 mL/kg of intravenous (IV) crystalloid fluid is recommended for adult septic patients within three hours of resuscitation. Balanced crystalloids have not only been associated with decreased mortality when compared to normal saline but have also been associated with a decreased risk of adverse effects, such as the hyperchloremic metabolic acidosis that often follows the administration of the sodium chloride-containing normal saline [9]. One study found that the administration of balanced crystalloids decreased mortality from any cause, including decreasing the chance of adverse kidney events, the incidence of new-renal replacement therapy, and persistent renal dysfunction [10].

Additionally, combined colloid and crystalloid therapy have been recommended by Surviving Sepsis Campaign Guidelines [11]. Specifically, the administration of albumin within 24 hours of crystalloids has been associated with a 28-day increased survival rate in septic adult patients (aged >18 years) [12].

In adults with septic shock, the use of peripheral vasopressors and corticosteroids can be recommended on a case-by-case basis, if fluid resuscitation fails to improve symptoms [13]. While there is no consensus on one particular steroid, one study shows that a combination of dexamethasone and methylprednisolone is more effective in reducing short-term sepsis mortality than either dexamethasone or methylprednisolone alone and that the administration of methylprednisolone improves ventilation-free days [13].

Moreover, other therapies seem to offer a promising future in terms of sepsis management. The anti-inflammatory effects of dexmedetomidine, a sedative and highly selective alpha-2 adrenergic agonist, has been studied in septic patients who required mechanical ventilation [14]. Results showed that although 14-day mortality did not improve in these patients, C-reactive protein, procalcitonin, and albumin levels all improved, highlighting the need for additional studies using larger sample sizes and higher doses [14]. While many studies are highlighting the potential benefits of these therapies in adult populations, more research needs to be conducted to determine whether they would be equally beneficial in the elderly adult populations, specifically.

Furthermore, sepsis is associated with several life-threatening sequelae, especially in the elderly. One study found that sepsis, male sex, being bedridden for >72 hours, history of venous thromboembolism (VTE), and mechanical ventilation were associated with VTE risk in elderly ICU patients [15]. This highlights the importance of VTE prophylaxis in septic patients, especially the elderly and those with complications that lead to the need for mechanical ventilation, such as ARDS. Unless there is a risk of significant bleeding or thrombocytopenia, low-molecular-weight heparin (LMWH) is the recommended VTE prophylactic agent in the intensive care setting [16].

Age is an independent risk factor for increased mortality in very elderly patients (aged >80 years) but not in the younger elderly (aged 65-79 years) [17]. In the very elderly patient population, administration of a resuscitation bundle within the first hours of hospital admission and prompt therapy within six hours of resuscitation has been associated with an improved mortality rate [17]. The sepsis resuscitation bundle consists of a variety of tasks to be completed within the first six hours of the sepsis presentation. These tasks include early blood cultures, empiric antibiotics, measuring central venous pressure, and central venous oxygen saturation as well as giving vasopressors to maintain the mean arterial pressure at or above 65 mmHg. If the arterial hypotension continues even with volume resuscitation or if their initial lactate was equal to or above 4 mmol/L for the first six hours, then the patient’s lactate levels were remeasured with a repeated focus examination [18].

Guidelines for ARDS management 

ARDS is defined by the acute onset of noncardiogenic pulmonary edema, hypoxemia, and the need for mechanical ventilation [4]. The majority of ARDS cases are caused by sepsis; however, other etiologies include pneumonia, trauma, viruses (respiratory syncytial virus and COVID-19), drug toxicity, and aspiration [19]. Risk factors for the development of ARDS include older age and direct and indirect lung injury [19]. Damage to the lungs or other organs is thought to trigger a release of inflammatory mediators, which can accumulate in the alveoli of the lungs. Inflammatory cell accumulation can cause damage to the vascular and alveolar epithelium, leading to pulmonary edema and hyaline membrane formation, which results in decreased lung compliance and gas exchange, manifesting as ARDS [19].

Diagnosis of ARDS is based on the presence of three Berlin criteria and is classified as mild, moderate, or severe from the oxygenation (measured by the ratio of partial pressure of arterial oxygen (PaO_2_) to a fraction of inspired oxygen (FiO2)). The international Berlin criteria to diagnose ARDS was established in 2011 in Berlin, where the European Society of Intensive Care Medicine, the American Thoracic Society, and the Society of Critical Care Medicine met to establish this new definition [19]. An ARDS diagnosis must present with (1) bilateral chest imaging opacities unexplained by nodules, effusions, or lung collapse; (2) pulmonary edema not explained by cardiac failure or fluid overload; and (3) symptom onset within one week of insult of respiratory symptoms. Once a patient presents with all three Berlin criteria, the severity is then ranked via O_2_ [19].

Management of ARDS relies on mechanical ventilation and is mainly supportive, aimed at eliminating the original cause, preventing progression and complications, and providing proper oxygenation and nutritional support [19]. Current guidelines for mechanical ventilation include maintaining an oxygen saturation of 88% to 95%, an arterial pH of 7.30 to 7.45, and a plateau pressure of no more than 30 cm H_2_O to avoid barotrauma [19]. Lower tidal volumes are preferred; however, patients must be closely watched and caution must be taken to avoid high partial pressures of arterial carbon dioxide (PaCO_2_). A PaCO_2_ of 50 mmHg or higher is independently associated with mortality [20]. For severe sepsis, mechanical ventilation in the prone position for more than 12 hours per day, which enhances oxygenation and lung recruitment, has been associated with reduced mortality. In this same patient demographic, high-frequency oscillatory ventilation is not routinely recommended, due to a lack of observed benefits and the potential for harm [21]. Additionally, studies have shown extracorporeal membrane oxygenation (ECMO) to be of benefit. ECMO uses a venoarterial or venovenous circuit to remove CO_2_ and introduce O_2_ before the blood returns to the body. ECMO may be used instead of mechanical ventilation, an alternative that allows patients to ambulate and participate in physical therapy, as well as receive oral nutrition [22]. However, according to the 2018 ECMO to Rescue Lung Injury in Severe ARDS (EOLIA) trial, ECMO was not found to improve the 60-day mortality rate in patients with severe ARDS, when compared to mechanical ventilation [23]. Personalized mechanical ventilation, an approach that takes into consideration the ARDS etiology, lung physiology and morphology, imaging, and biological phenotypes, may be an option for ARDS patients in the future; however, more research must be conducted before this strategy can be applied in clinical medicine [24].

Additionally, while not the routine form of treatment in ARDS, conservative fluid therapy and the use of diuretics have shown to be beneficial. A study conducted in 2016-2017 in Seattle, Washington, involved 234 adult patients who met moderate-to-severe ARDS criteria in the intensive care unit (ICU). Within 48-72 hours of meeting the criteria, half of the patients were given diuretics. In-hospital mortality was lower in the group that received diuretics than in the group that did not (14% vs. 25%; *P* = 0.025) [25].

Sepsis-induced ARDS prevention

The current approach to ARDS of any cause is supportive management. In ARDS caused by sepsis, the sepsis is treated until the patient develops ARDS, where at that point respiratory therapy will begin. However, within the past several years, there has been a shift in research toward preventative measures. Potential treatments that need to be considered while treating for sepsis to prevent ARDS are albumin infusion, bed elevation, and early antibiotics. Additionally, newer but promising techniques include using serum mannose as a preventative biomarker and targeting extracellular histones.

Albumin

As fluid management is a mainstay of sepsis management but also a potential cause of ARDS, the therapeutic balance may be difficult to achieve. With this in mind, albumin infusion should be highly considered due to its beneficial effects on ARDS and possibly toward sepsis. While the majority of current research specifies the need for additional studies, it has become clear that albumin may have a role as a niche drug in specific patient populations, such as those with ARDS and sepsis [26].

In the lungs, albumin promotes the maintenance of the glycocalyx layer, reduces inflammation, and improves alveolar-capillary membrane permeability [27]. A study of 993 ICU patients at the Vanderbilt University Medical Center concluded that lower serum albumin was independently associated with an increased risk of ARDS and that low plasma oncotic pressure contributes to pulmonary edema formation in patients at risk for ARDS within the first 72 hours after ICU admission [28]. With this information, it would be most beneficial to start treatment as soon as possible, especially within the first 72 hours. In addition, albumin is the main protein responsible for plasma colloid osmotic pressure, functioning as a transporter for endogenous and exogenous compounds and having both antioxidant and anti-inflammatory properties [29].

A recent study on sepsis treatment compared the use of crystalloids combined with albumin to crystalloids alone. The results showed that when patients received albumin within the first 24 hours, the combination albumin therapy was associated with a decreased 28-day mortality [12]. Additionally, a similar study demonstrated a trend toward reduced 90-day mortality in patients with severe sepsis given albumin when compared to crystalloid and saline solutions [30]. More studies need to be conducted as the beneficial results have not been replicable and some studies show no benefit in sepsis [29].

There are currently no studies to show if albumin treatment during sepsis can improve lung health and prevent the development of ARDS. Thus, future studies are needed to further explore the role of albumin in ARDS prophylaxis.

Bed Elevation

Aspiration of gastric contents into the lower respiratory tract, which is common in critically ill patients, is an independent risk factor for the development of ARDS [31]. However, placing patients in a semi-recumbent position, with the head of the bed raised to 30-45 degrees reduces the risk of tracheal aspiration and ventilator pneumonia, particularly when mental state is impaired or enteral nutrition is administered [32]. Compliance with proper bed angle has proved difficult, leading to various studies on head-of-bed perception by medical staff. One study at a level I trauma center in Seattle, Washington, tested the head-of-bed angle perception of 175 clinicians (nurses, physicians, respiratory therapists, physical and occupational therapists, and medical and hospital assistants). The results showed that 50% to 86% of these clinicians preserve head-of-bed angles correctly. It showed that nurses tended to underestimate the angle of the bed, while other types of clinicians tended to overestimate [33]. Various bed systems are being developed, which indicate when the head-of-bed angle drops below 30 degrees, increasing the risk for pneumonia. A four-week trial of a bed system device showed that out of 268-bed measurements, the average head-of-bed elevation was 21.8 degrees on beds without the device (*n* = 166) and 30.9 degrees on beds with the device (*n* = 102; *P* < 0.005) [34]. Improved accuracy in head-of-bed elevation may prove to be a simple and effective technique to prevent ARDS.

Future considerations for sepsis-induced ARDS prevention

When pulmonary edema and hyaline membrane formation occur due to increased inflammatory mediators during sepsis, patients experience a decrease in lung compliance and gas exchange [19]. New research in the successful management of the sepsis inflammatory response shows benefits toward ARDS prevention.

Serum Mannose Biomarkers

Recent research demonstrates that genetically regulated serum mannose is associated with ARDS risk and outcome, whereas increased serum mannose upon admission was associated with reduced ARDS risk and better survival rate [35]. Using mannose as a biomarker for sepsis patients could lead to prevention and clinical intervention in ARDS cases [35].

Extracellular Histones Therapy

Recent studies have shown extracellular histones to be an inflammatory mediator that plays an important role in the pathogenesis of both sepsis and ARDS [36,37]. Histones are intranuclear proteins. However, in sepsis, significant cellular death results in the release of histones into the blood. Extracellular histones then act as damage-associated molecular pattern proteins, activating the immune system and causing further cytotoxicity [38]. They cause endothelial injury and dysfunction, hemorrhage, thrombosis, and organ failure [37]. Plasma from ARDS patients revealed elevated histones in nonsurvivors compared to survivors [36].

Targeting histones through neutralizing antibodies or heparin substantiates potent protective effects, suggesting a potential therapeutic strategy for sepsis and the prevention of ARDS [36]. The effect of small polyanions (SPAs) and heparin in neutralizing histones have been studied in animal models. SPAs interact electrostatically with histones to neutralize their pathological effects [39]. SPAs have been shown to significantly inhibit sepsis, deep-vein thrombosis, and cardiac and tissue-flap models of ischemia-reperfusion injury (IRI) in mice and rats. Heparin has been shown to bind extracellular histones and provide cytoprotection in mouse models of sterile inflammation and sepsis [40]. Further clinical trials are needed to evaluate the beneficial impact SPAs and heparin may have in the prevention of ARDS in sepsis.

Osteopathic considerations

Osteopathic manipulative treatment (OMT) aims to treat somatic dysfunctions noninvasively by evaluating the body, mind, and spirit as one unit, assessing the function of anatomical structure, improving blood circulation, and encouraging the body to self-heal. Osteopathic principles are known for their holistic approach to patient care about the diagnosis and treatment of disease. In a study done on hospitalized patients, the patients reported that OMT both improved their overall comfort level (98%) and reduced their anxiety (90%) and pain (74%) [41]. Additionally, 98% of surveyed patients would recommend OMT to other hospitalized patients [41]. In identifying possible preventative measures to treat patients with sepsis and sepsis-induced ARDS, the use of OMT as an adjunct therapy should be considered. Currently, there are no publications that explore the use of OMT in sepsis and ARDS. However, there are some publications on the use of OMT in pneumonia and chronic obstructive pulmonary disease (COPD)-associated ARDS. A summary of these findings is given in Table 1.

**Table 1:** Effectiveness of various OMT techniques in pneumonia treatment. OMT, osteopathic manipulative treatment

Respiratory disease	OMT techniques	Results
Pneumonia	Thoracolumbar soft tissue	Alleviate patient discomfort [42]
	Rib raising	Reduced length of hospital stay (aged 50-74 years) [42]
	Doming of the diaphragm with myofascial release	Reduced hospital stay by two days [43]
	Thoracic lymphatic pump	Reduced rate of respiratory distress by 8% [42]
	Pedal pump	Lowered hospital mortality rate by 6% in adult patients and 11% in elderly patients (aged ≥75 years) [42]

One study on pneumonia found that thoracolumbar soft tissue, rib raising, doming of the diaphragm with myofascial release, cervical spine soft tissue, suboccipital decompression, thoracic inlet myofascial release, thoracic lymphatic pump, and pedal pump, are osteopathic techniques that alleviate the patient's discomfort, reduce the length of hospital stay (aged 50-74 years), and lower in-hospital mortality rates (aged ≥70 years). The study also reported that OMT reduced the rate of respiratory distress by 8% in pneumonia patients and lowered the hospital mortality rate by 6% in adult patients and 11% in elderly patients (aged ≥75 years) [42]. Another study also found that adding OMT as an adjunct therapy to conventional pneumonia treatment reduces the length of hospital stay by two days [43]. Similarly, OMT is beneficial in ARDS due to COPD. It has also been shown that OMT evaluation and management were beneficial to reducing the blood gas impairment recovery period and improving the total lung capacity in this population [44].

Due to the physiologic nature of ARDS or sepsis-induced ARDS, it may be beneficial for osteopathic physicians to focus treatment on alleviating rib and thoracic somatic dysfunctions. Normal physiological respiration requires movement of the ribs, sternum, and diaphragm to create and maintain intra-abdominal pressure differences. When the respiratory system is dysfunctional, oxygen exchange decreases, leading to further, and direr, complications, including death. Additionally, untreated rib dysfunctions may lead to musculoskeletal pain [45]. To improve lung and breathing functionality, the osteopath’s goal is to ensure that the diaphragm and ribs have the adequate space needed to move freely, improving the patient’s homeostatic processes and stimulating intrinsic self-healing [45,46].

It is also important to consider possible contraindications to OMT in this patient population. The use of lymph flow OMT techniques are contraindicated on patients with bacterial infections [47], such as in bacterial sepsis-induced ARDS. Additionally, it is also important to consider that OMT is contraindicated in patients with bacterial infections and a current temperature over 102 °F [48].

OMT techniques that treat rib and thoracic dysfunctions may be beneficial for patients with ARDS or sepsis-induced ARDS, although more research is needed to validate this claim.

## Conclusions

The goal of this paper was to review the current guidelines for both sepsis and ARDS to determine preventative management for sepsis-induced ARDS. Various techniques can be applied when diagnosing and treating elderly patients with sepsis to prevent the onset of ARDS. Techniques vary in their complexity, with simpler treatment options, including bed elevation and early antibiotics. Additionally, albumin infusion may be beneficial; however, more research must be conducted. Inflammatory mediators, including serum mannose biomarkers and extracellular histone therapy, show a promising avenue for future treatment. Currently, little to no research has been done on the use of OMT on ARDS or sepsis-induced ARDS. OMT, which focuses on alleviating rib and thoracic somatic dysfunctions, has been used as an adjunct therapy to treat other respiratory diseases, such as pneumonia and COPD. The results of these studies may garner interest in whether the use of OMT as an adjunct therapy may be beneficial for patients with ARDS or sepsis-induced ARDS. Research is needed to assess the effectiveness of OMT in treating patients with ARDS or sepsis-induced ARDS.
